# Perceived Facilitators and Barriers, From the Perspective of Users, of a Multicomponent Intervention in Older People Using an Asynchronous Telehealth Modality During the COVID-19 Pandemic: A Qualitative Research

**DOI:** 10.1155/jare/6839569

**Published:** 2025-03-31

**Authors:** Rafael Pizarro-Mena, Samuel Duran-Aguero, Maria Causa-Vera, Camilo Rios-Duran, Solange Parra-Soto

**Affiliations:** ^1^Facultad de Ciencias de la Rehabilitación y Calidad de Vida, Universidad San Sebastián, Sede Los Leones, Santiago, Chile; ^2^Dirección de Vinculación con el Medio, Vicerrectoría de Estudiantes y Vinculación con el Medio, Universidad Santo Tomás, Chile; ^3^Programa MAS Adultos Mayores Autovalentes (+AMA), San Joaquín, Chile; ^4^Departamento de Nutrición y Salud Pública, Facultad Ciencias de la Salud y de los Alimentos, Universidad del Bío-Bío, Chillán, Chile

**Keywords:** barriers, COVID-19, facilitators, multicomponent, older people, telehealth

## Abstract

**Objective:** The COVID-19 pandemic interrupted multicomponent face-to-face interventions with older people, which became an opportunity for the implementation of these interventions in telehealth modality, as well as the analysis of the facilitators and barriers. This qualitative study examines the facilitators and barriers, from the users' perspective, of a promotional–preventive multicomponent intervention in older people using an asynchronous telehealth modality during the COVID-19 pandemic, as a continuation of the face-to-face intervention.

**Methods:** Semistructured in-depth interviews were used. An intentional sampling was conducted over eight groups of older people in a city, who were part of a multicomponent (physical activity, cognitive stimulation, and education) telehealth (videos, infographics, manual, and WhatsApp) promotional–preventive intervention, who came from the same face-to-face intervention. After intervention, two groups were identified (intervention and control). Telephone interviews, until theoretical saturation was reached, were audio-recorded and transcribed. Thematic analysis was conducted using Atlas.ti.

**Results:** Twenty-six older people of both sexes, aged 60–88 years, were interviewed (14 intervened and 12 controls). Six themes were identified: positive aspects of telehealth, telehealth facilitators, preference for face-to-face modality over telehealth modality, telehealth barriers, reasons for not performing telehealth, and coping strategies in the pandemic: specifically, as facilitators, participating in the company of others, having participated in the same intervention previously (face-to-face modality), good knowledge of digital literacy, self-motivation, commitment to the program, and the emergence of innate leaders, and as barriers, pain during physical activity, complexity of cognitive exercises included in cognitive stimulation, poor digital literacy, and not having support from others.

**Conclusion:** This is the first qualitative study that identifies facilitators and barriers of a multicomponent intervention in an asynchronous telehealth modality, as a continuation of the same face-to-face intervention. The asynchronous telehealth modality could be used regularly with older people in rural areas, in situations of disability and/or with care needs, pandemic scenarios, or natural disasters.

## 1. Introduction

The World Health Organization (WHO) declared COVID-19 a “pandemic,” which led to quarantines and restrictions on gatherings, particularly for older people, negatively impacting their health, resulting in a geriatric emergency [1], associated with physical inactivity [2] and mental health problems [3]. In addition, it affected the continuity of regular governmental interventions, due to the lack of accessibility [4], as in the case of the more self-reliant older adults program (+AMA, as per its acronym in Spanish [programa Más Adultos Mayores Autovalentes]) [5]. The pandemic also interrupted diverse research works, which forced their suspension or the implementation of remote contactless methods [6], providing a research opportunity for the application of telehealth.

The +AMA program is a nonpharmacological multicomponent intervention [7, 8] of primary healthcare, with a promotional–preventive emphasis, implemented by the Chilean Ministry of Health [5]. This multicomponent intervention aims to maintain or improve functionality and quality of life of older people; it consists of three components: physical activity, cognitive stimulation, and education; it is carried out in mixed sessions, that is, physical activity + cognitive stimulation or physical activity + education, two times a week. There are a total of 24 mixed sessions, carried out during a period of 3 months, and are performed once a year. The intervention is carried out in groups of people aged 60 and over, in groups of 15–20 older people (groups diverse in sex, age, education, and seniority in the program); these groups are in different cities of the country (in urban and rural contexts). Depending on the size of the city and the number of primary care centers, in the same city there may be several groups intervened in parallel. The intervention is carried out by a professional pair (kinesiologist and occupational therapist/speech and language therapist). Only the effects of this intervention are evaluated in terms of management identifiers and not through the spheres of the Comprehensive Gerontological Assessment. Since the beginning of this public policy in 2015, around 500 professional pairs have been implemented nationwide, and this multicomponent intervention is carried out regularly every year [5]. In addition, professionals train and monitor older people and community leaders, in the three interventions so that they can continue these interventions in the months when the +AMA program is not carried out.

In the pandemic context, it has been described that multicomponent interventions positively affect biopsychosocial health [9] since they mitigate the effects on the mental health of older people, the reason why they should be implemented and/or continued. In addition, the WHO, in order to keep activities of daily living, has encouraged the maintenance of virtual–social activities and regular engagement in physical activity at home [10]. In addition, the main international recommendations have been oriented toward designing and implementing cognitive strategies and increasing levels of physical activity, using applications, online videos, and telehealth [11]. Moreover, the adoption of available and underused services and products, such as telehealth, was accelerated and normalized [6], transforming it into a new modality of intervention that ensures availability and access to social and health services in older people [12].

Telehealth is understood as a modality that makes use of information and communication technologies (ICTs) to provide health services, healthcare, and information, regardless of distance, placing emphasis on prevention. There are three modalities that may be applied: synchronous (real-time interaction between the professional and the user), asynchronous (not simultaneous or concurrent interaction), and hybrid (a combination of both) [13]. A review found that there is an increasing interest and uptake of telehealth by older people since the outbreak of the pandemic because when it is provided in a friendly and collaborative manner, older people show a greater uptake [14], with several experiences having been collected to this date [15–17]. However, telehealth in older people has been focused on curative services and is mostly concentrated in the Northern Hemisphere or high-income countries [14], with greater emphasis on research from the point of view of healthcare providers, during the pandemic [18, 19]. Few studies provide a response to questions about the experience, facilitators, and barriers [14] from the perspective of older people themselves, factors that may differ among who participated in the intervention and who did not, with these differences being useful for identifying special needs when designing and implementing interventions.

The coverage model effective [20] is useful to analyze equity in access to healthcare because it facilitates identification of specific groups with unmet needs and identifies barriers and facilitators that hinder or favor the achievement of effective coverage in each of those groups. It considers stages in the access process to obtain effective coverage: availability, accessibility, acceptability, contact, and effectiveness coverage.

In Chile, different organizations have developed best practices to implement telehealth [13], with Chile being one of the most advanced in the Latin American region. With the onset of the pandemic, the Chilean Ministry of Health recommended the team of the +AMA program to conduct the intervention in the telehealth modality (synchronous, asynchronous, and/or with the aid of manuals) [5]. Therefore, the objective of this qualitative research was to identify the perceived facilitators and barriers, from the users' perspective, of a promotional–preventive multicomponent intervention in older people using an asynchronous telehealth modality during the COVID-19 pandemic, as a continuation of the face-to-face intervention.

## 2. Methods

### 2.1. Design

A qualitative design and the technique of in-depth semistructured interviews were used. This manuscript has been compiled in accordance with the Consolidated Criteria for Reporting Qualitative Research (COREQ) 32-item checklist [21], which is found in the material supporting 1.

### 2.2. Sampling and Participants

An intentional sampling was conducted over eight groups of older people from a city in the Metropolitan Region of Chile, with a medium–low socioeconomic level, who would take part in a multicomponent intervention in primary healthcare and who would begin with the multicomponent intervention (+AMA) in March 2020. This intervention lasts 3 months each year, and it has been carried out regularly for the last 5 years since its initial implementation in 2015 as a national public policy. This, in order to identify the effects in the health of this face-to-face intervention, is based on the experience of the older people, including shared dimensions and/or several variations. To reach theoretical saturation, a sampling framework was prospectively developed to seek out participants from at least five of the eight groups, whose sex, age, level of education, and seniority in the program would be diverse, obtaining a representative set of data.

In the scenario of COVID-19 lockdown and with the restrictions on in-person gatherings imposed by the sanitary authority, in March 2020 the intervention shifted from a face-to-face modality to an asynchronous telehealth modality, to implement the program at a local level, for a period of 8 months. Prior to the pandemic, these older people had not participated in any telehealth or digital literacy interventions. Since it was a local initiative, it became a pilot pioneering intervention at a national level. After this period was completed, two groups were identified: the intervention group (IG), who were subjected to the intervention, and the control group (CG), who did not intervene, representing the five groups originally projected.

Sample selection criteria were as follows: (a) older people aged 60 years and older of both sexes, (b) self-reliant, (c) able to see and hear well enough to participate in the intervention, and (d) without a physical and/or cognitive disease/disability that will affect participation. In addition, a criterion was added for the IG: they must have participated in the multicomponent telehealth intervention for 8 months, and for the CG: they must not have been part of a similar intervention during the follow-up period and performing regular controls in primary healthcare.

**Figure 1:**
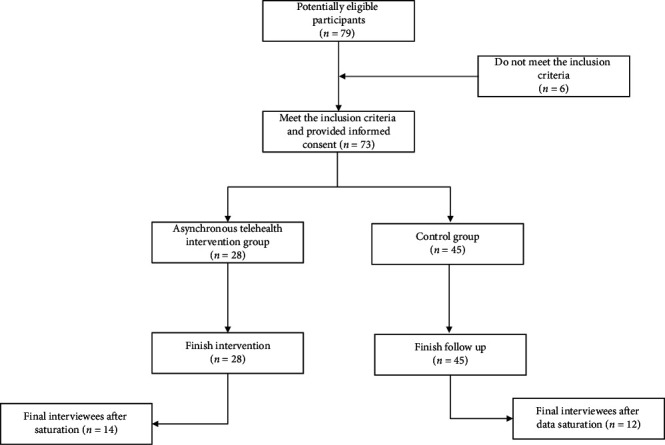
Recruitment flow diagram.

Then, in-depth semistructured interviews were conducted in both groups. During the process of data collection, the research team identified theoretical saturation [22], and consequently, the interviews were stopped. The recruitment flow diagram is presented in Figure 1.

### 2.3. Procedure

#### 2.3.1. Intervention

**Figure 2:**
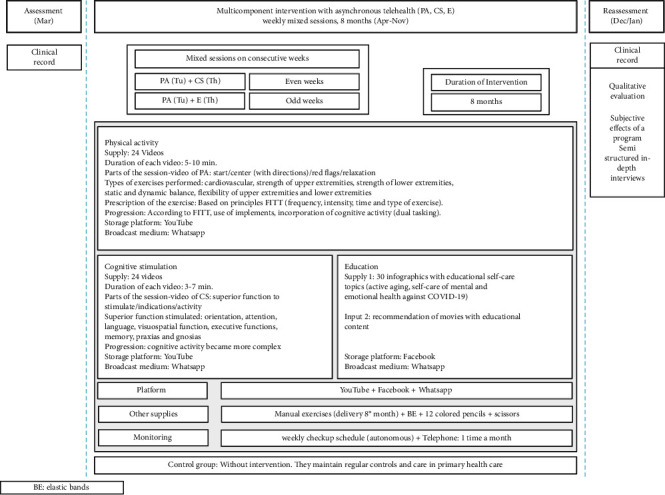
Protocol of the multicomponent intervention in asynchronous telehealth modality.

The multicomponent intervention in an asynchronous telehealth modality was designed and implemented by kinesiologists and occupational therapists with 5 years of experience and training in the application of face-to-face intervention. The multicomponent intervention consisted of three interventions/components: physical activity, cognitive stimulation, and education. For 8 months (April–November 2020), participants were instructed to perform weekly consecutive mixed activities (a week of physical activity and cognitive stimulation and another week of physical activity and education). The team created videos that were stored on YouTube and infographics that were stored on Facebook; this content was disseminated through WhatsApp every week and sent to the five groups of older people for them to perform the activities. Telephone monitoring was performed once a month. Additionally, during the last month of the intervention, older people were personally handed a manual containing the same exercises/activities (a weekly checkup schedule was included), along with other implements. In this way, telehealth intervention was asynchronous during the pandemic, with only the methodology (from the modality face-to-face to telehealth modality) and the time of intervention (from 3 to 8 months) being modified to protect older people during the pandemic, and without changing neither the objective nor the activities included in the face-to-face intervention conducted in previous years. Once the intervention was completed, two groups were identified (IG and CG). The intervention protocol and characteristics of each component are presented (Figure 2).

Prior to the interviews, and in order to identify the characteristics of the multicomponent intervention conducted with the 28 older people who participated in the asynchronous telehealth modality, a structured telephone survey was administered. The survey addressed the weekly frequency and duration of each component. Additionally, for physical activity, two more variables were assessed (type and intensity of exercise), which align with the FITT principle for physical exercise (frequency, intensity, type, and time) [23]. The results are presented in Table 1.

#### 2.3.2. Interviews

Two in-depth semistructured interview guidelines (IG and CG) were applied via telephone while older people were at home. The guidelines, based on the existing literature and related to the effects, barriers, and facilitators of telehealth and multicomponent interventions in older people [5, 7, 24], included nontargeted open-ended questions, addressing similar topics and with some differences previously defined, with the purpose of meeting the research questions, and ensuring that interviews ended positively.

The main researcher is a PhD in Gerontology Research with more than 20 years of academic work on undergraduate and postgraduate gerontology-related programs, who worked as a university professor when the research was conducted, and also, with experience carried out in similar studies. This researcher, established a triangular cooperation with other three researchers to define the content, order and delivery of the questions, ensuring to incorporate probes, and a plain and understandable language for older people. One-time interviews were conducted by the main researcher (January 2021), who only knew these older people when the informed consent was obtained 10 months earlier, and without interference from the team that carried out the intervention, to achieve greater objectivity; the interviews lasted between 30 and 60 min and were audio-recorded. In addition, field notes and audio reflections were added after the interviews. At the end of each interview, we proceeded to reflect with older people on the effects, achieved or possible to achieve, secondary to the intervention in face-to-face, and telehealth modalities. Sociodemographic information was recorded: sex, age, educational level, and years taking part in the face-to-face intervention (antiquity).

#### 2.3.3. Data Analysis

**Figure 3:**
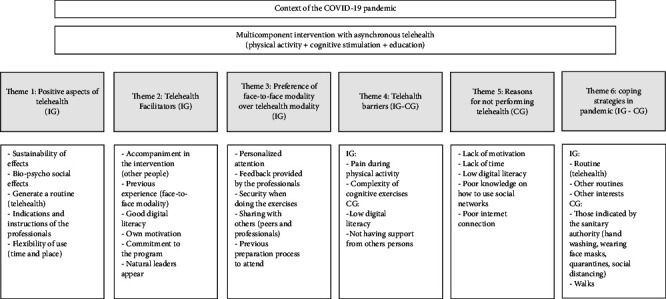
Diagram illustrating themes.

**Figure 4:**
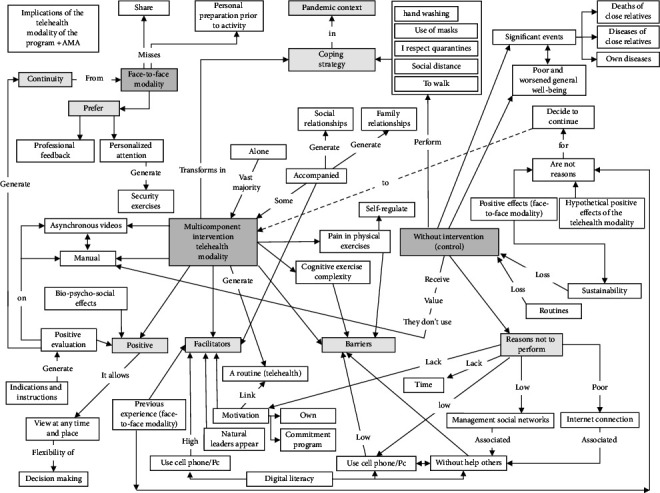
Scheme summarizing the implications of the multicomponent intervention in the asynchronous telehealth modality, on the basis of reported information provided by older people (intervention group and control group).

Thematic analysis was used to report patterns [25]. All the interviews were entirely transcribed by a researcher. The transcripts were imported into the Atlas.ti software. It was followed over a five-stage process [25]: (1) familiarization of transcripts, (2) generating recurrent data into codes, (3) collating codes into themes, (4) reviewing of themes, and (5) refinement of themes, in an iterative–reflective process. The principal investigator reviewed the coding and the final thematic structure, verifying its consistency. Once the thematic structure had been completed, the researcher–sociologist independently encoded three transcripts using the final coding framework. A realist epistemological approach was assumed; an inductive approach was used in analysis with data-driven codes and themes; the four researchers (multidisciplinary team) carried out an interpretive analysis process, triangulating to avoid bias toward a particular approach; the meanings resulted in themes and subthemes [25], based on the statements of the participants; this ensures that they are relevant to the study topic and congruent with older people viewpoints. A sufficient degree of internal homogeneity was explored so the quotes assigned to the same theme were clearly related and the external heterogeneity ensured quotes assigned to different subthemes were different from each other [26]. Older people confirmed the findings, and a diagram of themes and subthemes was made (Figures 3 and 4).

#### 2.3.4. Ethical Approval and Consent to Participate

The study was approved by the Ethics Committee of the South Metropolitan Health Service (Servicio de Salud Metropolitano Sur) (04-07112019). After having read the informed consent, all older people agreed to sign voluntarily, before the intervention and the interviews were conducted.

## 3. Results

Of the 79 older people who initially participated in the intervention (five out of the eight groups of older people), 73 were eligible at the end (28 older people in IG and 45 in CG). Afterward, 14 older people of IG and 12 older people of CG (Table 2) were interviewed in depth. Six themes were identified.

### 3.1. Theme 1: Positive Aspects of Telehealth

Older people who participated in the intervention in the telehealth modality during the pandemic (IG) transitioned from the same face-to-face intervention (in previous years) and reported a diverse array of biopsychosocial positive effects that were achieved in the face-to-face modality and were sustained over time with the telehealth. A routine was developed based on telehealth, transforming it into a coping strategy to face the pandemic. Most older people who engaged in the intervention participated on their own. A smaller group had the company of others, which resulted in the creation of social and family relationships.

*“They are easy to understand and when I find it very hard to use the phone, my grandson helps me and I ask him for help, he teaches me the same over and over again, he explains again so I can understand well”* (IG-I6, female, 80 years old).

Older people from the IG reported a positive evaluation of telehealth modality, due to the indications and instructions of the professionals.

*“They make us use different things for made the exercise, as a dumbbell, like a kilo of rice, a liter of water, so you see the possibilities that you have at home”* (IG-I6, female, 80 years old).

In addition, they appreciated that the videos/manual enabled them to watch the content anytime and anywhere, which leads to flexibility in decision-making.

*“I have it at home and can repeat it the times that I want (video), and well, they're kind of like the same that we did (in the face-to-face modality)…. You don't stop exercising when you were supposed to stop and there you have them, available for you”* (IG-I7, female, 60 years old).

*“With the book (manual) you can do them at any time, you can make up a routine yourself”* (IG-I13, female, 65 years old).

### 3.2. Theme 2: Telehealth Facilitators

Older people from the IG mentioned as facilitators the fact of participating in the telehealth intervention in the company of others, the experience of having participated in the same intervention previously (face-to-face modality), and having a good knowledge of digital literacy, that is, high engagement in cell phone/personal computer use.

*“We usually do it together (with his wife) at the same time watching the screen and sometimes when there are exercises when you need assistance from others…she helps me a little bit”* (IG-I10, male, 67 years old).

*“The kinesiologists had already made us do most of those exercises in the weekly workshops last year (face-to-face modality)”* (IG-I11, male, 75 years old).

Additionally, self-motivation, commitment to the program, and the emergence of innate leaders are facilitators. Motivation is linked to the routine established with this modality.

*“It's a motivation, because I share it with other people who are not part of the workshop…I send it to other friends who are alone at home*” (IG-I6, female, 80 years old).

### 3.3. Theme 3: Preference for Face-to-Face Modality Over Telehealth Modality

Older people from the IG preferred the face-to-face modality over telehealth because of the personalized attention and feedback provided by the professionals, which generated security at the moment of doing the exercises.

*“I prefer the face-to-face workshop, because you don't know how far you can go (in Telehealth modality). They told us: do it as you can, if you can't touch the floor, then touch your knees; and if you can't, touch your calves (in the face-to-face modality)”* (IG-I14, female, 60 years old).

In addition, they missed sharing with other older people and with the professionals, as well as the process of preparation that the in-person activity entailed.

*“Face-to-face is better than the video, the handbook, because of the human contact with others. That's priceless, it's kind of a benefit besides the physical exercise, the communication with others has an immeasurable value”* (IG-I5, female, 69 years old).

### 3.4. Theme 4: Telehealth Barriers

Older people from the intervention group identified the pain during physical activity, which they have learned to self-regulate thanks to the learning and education obtained in the intervention in face-to-face modality made in previous years (prior to the pandemic), and the complexity of cognitive exercises included in the component of cognitive stimulation.

*“Doing squats makes my knees hurt, but when they hurt, I just do less reps”* (IG-I4, female, 80 years old).

*“The one we did the other time, we had to remember a shape in different positions. Then they asked in what position was the first, what's the position of the other, and that was the most difficult for me”* (IG-I8, female, 68 years old).

Older people from the CG identified poor digital literacy due to low cell phone and/or computer skills and not having support from others. Both barriers were associated.

*“They had told us, the ones from the exercises (professionals), that they were going to teach us how to use the phones. Anyway, with this virus issue it couldn't be done, and I don't have anyone to teach me, because I live alone”* (CG-I1, female, 73 years old).

### 3.5. Theme 5: Reasons for Not Performing Telehealth

Older people from the CG have the experience of having participated in the face-to-face intervention previously; they know the biological, psychoaffective, and social effects achieved in that intervention and identify/hypothesize the potential biopsychosocial positive effects of telehealth. Nevertheless, they decided not to transform from one modality to another, which resulted in the effects not being sustained, the loss of routines, and not generating a new routine (telehealth) during the pandemic.

*“Yes, I think they would have helped me. I'm watching those who are doing the exercises, and I'd feel better doing the exercises together with them”* (CG-I5, female, 75 years old).

These older people mentioned as reasons the lack of motivation or time, low engagement in cell phone/computer use, poor knowledge on how to use social networks, and poor Internet connection; the last two reasons are associated with the fact of not having someone to help them.

*“You don't do exercise if you're not given directions, and if you don't have other people doing the exercises with you. There's no motivation in doing it alone. Unless it's a person who has made exercise their entire life”* (CG-I12, male, 73 years old).

*“I don't have internet. ‘Cause now we can't meet other people that much, then it's hard to go somewhere and say, well, let's watch the video”* (CG-I4, female, 62 years old).

This same group of older people were sent videos through WhatsApp groups and were handed the manual in person; this manual was appreciated but not used.

*“The manual, I loved it, in the first place because of the exercises and in the second place, because the young men (professionals) that were working with us appear in it…So, the fact of seeing them, even if it's just in pictures, is pleasing”* (CG-I9, female, 71 years old).

*“Very good (the manual), it's just that I haven't solved any of the problems that appear there, just for being lazy. Because I know how to solve them all and I understand them…they're the same we did before there (face-to-face modality)”* (CG-I7, male, 79 years old).

### 3.6. Theme 6: Coping Strategies in Pandemic

Older people from the CG used the measures adopted by the sanitary authority as a coping strategy: hand washing, wearing face masks, respecting quarantines, and social distancing. In addition, they took walks since they remembered the educational content provided when they engaged in the physical activity component in the face-to-face modality in previous years.

*“It's very important wearing the mask, hand-washing…one's hygiene”* (CG-I5, female, 75 years old).

*“Every night I take a walk…I walk for 10 minutes, sometimes I walk up to half an hour”* (CG-I1, female, 73 years old).

Noteworthy is that they do not mention engaging in physical activity and/or cognitive stimulation on their own initiative as a coping strategy although they learned them during the face-to-face intervention.

Older people from the CG showed a poor, more impaired general well-being, with significant events such as the death of close others, and diseases affecting them or affecting close others.

*“The legs were the most benefited (face-to-face modality). Now I don't feel very well. My legs hurt, my knees…It's a little hard to walk”* (CG-I3, male, 88 years old).

*“It's been difficult because I've been very depressive, crying a lot”* (CG-I4, female, 62 years old).

*“First it was my mother, she fell ill in March, in April my husband had a stroke”* (CG-I10, female, 69 years old).

Coping strategies were different in the IG that presented a diverse array of positive biological, psychoaffective, and social effects, which were sustained since the face-to-face modality, or new effects produced by telehealth. Moreover, older people from the IG have found a new routine with telehealth and have developed other routines or interests, and their mood state has remained unaffected during the pandemic.

*“I think they are good for everything, for the body, for the soul, for the mind”* (IG-I4, female, 80 years old).

## 4. Discussion

The main result of our investigation is that, from the experience of older people, after an 8-month multicomponent promotional–preventive (physical activity + cognitive stimulation + education) intervention in the telehealth modality, as a continuation of the same face-to-face intervention, they reported positive aspects, facilitators, barriers, and preferences over the telehealth modality, this being a strategy for coping with the pandemic. In addition, they reported reasons for not performing telehealth.

The multicomponent interventions (complex, multimodal, multidomain, or multidimensional) [7, 8] consist of a series of nonpharmacological, behavioral, and environmental strategies [27] that combine multiple health promotion activities [28], being highlighted among their components: cognitive stimulation, physical activity, education, management of risk factors, nutrition and caloric restriction, optimization of stress levels and psychological well-being, leisure activities, and social support [29, 30]. It has been noted that these interventions are more beneficial than a single intervention (one component), due to a possible synergistic effect of various components [31].

In older people of the community, these multicomponent interventions, which include diverse combinations of its components, have been focused on daily functioning [32], the reduction of risk factors and chronic diseases [33], the prevention of falls [34], the cognitive deterioration and dementia [30], the frailty [35], and the multidimensional frailty [36]. In addition, they lead to improvements in physical, psychological, cognitive, and social functioning, promoting healthy aging [7], increasing healthy life expectancy [36], therefore prolonging cognitive life associated with normal aging [29], and improving brain health [27], thus reflecting that multicomponent interventions have several effects on biopsychosocial health and quality of life of older people.

Similarly, these multicomponent interventions have been developed as part of public policies in some countries; this is the case of the +AMA program [5]. The COVID-19 pandemic in 2020, and secondary quarantines and restrictions on gatherings, also affected the implementation of interventions and the continuity of others that already existed [4], as it happened with the +AMA program.

Quarantines and restrictions on gatherings, when applied as public health measures, have failed to consider heterogeneity, life stories, and different forms of aging of older people [1], going from physical distancing to social distancing. They are an unpleasant experience because of separation from loved ones, the loss of freedom, boredom, and uncertainty over health. However, telehealth has enabled the continuity of care services and avoided the risk of contagion [14, 37]. In addition, older people have increased the use of telehealth and ICTs and have learned new technologies more quickly [38], allowing them to continue with their activities of daily living, exchanging informal support from family and neighbors and formal support from community organizations, and keeping themselves physically active, as part of their resilience [39]. Therefore, the pandemic has offered an opportunity to adapt the current delivery systems of social and health services for older people; and consequently, the design, implementation, evaluation and intervention strategies, of a face-to-face nature, benefited from the Telehealth modality to continue with the face-to-face intervention modalities, they will benefit from the telehealth modality to continue face-to-face intervention.

The profile of older people who participated in telehealth and interviews in this study primarily consisted of women aged 80 years or younger, residing in a city of the metropolitan region, belonging to a medium–low socioeconomic stratum, and regularly attending a government-run primary healthcare program. These individuals experienced disruptions in the continuity of their prior in-person intervention due to the pandemic.

In this context, it has been documented that older women under 85 years of age, living in major cities, cohabiting with others, belonging to socioeconomically disadvantaged areas, and having disabilities, chronic illnesses, multimorbidity, lower perceived quality of life, or experiencing delays or interruptions in healthcare access, higher levels of distress, and/or reduced loneliness were more likely to use telehealth services during the COVID-19 pandemic [40].

Regarding facilitators, in a face-to-face intervention focused on the prevention of falls [41], older people mentioned as facilitators the content and guidelines of the intervention, which are crucial for generating adherence and continuity. They also mentioned that the intervention was targeted at older people, it was feasible, they obtained more than they expected, their achievements were acknowledged, and they had a good time and became enthusiastic; in addition, they also mentioned that the instructors were enthusiastic, promoted socialization, and provided personalized support [41]. Several of these facilitators were identified in our research, which would reflect that facilitators of face-to-face modalities are also found in telehealth and allow for the sustainability of interventions. Beyond the fact of sharing with fellow older people, one of the aspects they missed the most from the face-to-face modality was the feedback and personalized attention given by the professionals when performing physical activity [41]; this facilitator is not identified in the asynchronous telehealth modality and should be promoted in future telehealth interventions.

Other facilitators of telehealth identified in our research, and also recently reported, include having support networks, access to technology and the Internet, and the availability of devices and resources that enable participation. These factors significantly enhance the feasibility of telehealth interventions by reducing barriers and fostering a more inclusive approach to healthcare for older people [42]. Additionally, one of the strategies that this government intervention uses in person is the training of monitoring older people and community leaders and providing continuity to the intervention in the community [5]. One of the facilitators identified in our study was the emergence of some natural leaders. It is possible that this experience from previous years appeared spontaneously during telehealth, without any further intention on the part of professionals. It is possible that after 5 years of carrying out the intervention (every year), it was in the collective memory of older people. This presents the opportunity to identify and train them, so they can support professionals in the execution of interventions with a promotional focus and can support other older people during telehealth.

One of the barriers we identified particularly in telehealth is the pain produced by physical activity and the complexity of cognitive exercises (cognitive stimulation), which provide new evidence, and from an overall perspective, several barriers are in line with previous findings. In this context, and more recently, from the perspective of older people, barriers to telehealth have been described, including low digital literacy, complicated application interfaces, and limited support networks (the latter being one of the most significant factors) [42]. These barriers are often interconnected as individuals with limited support networks are less likely to receive the assistance needed to navigate digital platforms. Additionally, many older people express a preference for in-person interventions, highlighting the challenges of fully transitioning to virtual healthcare models [42]. In addition, among the barriers to Telehealth (video), from the perspective of providers, they mentioned low cognitive and sensory abilities, technology access, and reliance on caregivers [43]; older age and low income were also negatively associated with the use of telehealth; nevertheless, when enabling factors were included (having a cell phone/computer/tablet, knowledge of how to use them, and having Internet access), these barriers were no longer significant [12], and therefore, it would be relevant to provide economic resources (Internet data packs) in future studies and interventions.

In this sense, commuting problems, such as place, frequency, and schedule of transportation, have been identified among the barriers to carrying on with face-to-face interventions [41]; this could be avoided in asynchronous telehealth since as we mentioned before this modality allows older people to engage in the intervention anytime and anywhere, which leads to flexibility and autonomy.

The reasons for not engaging in telehealth are a topic that has been scarcely addressed in research, as indicated recently [42]. Therefore, our study provides new evidence by analyzing two groups of older people: those who continued with telehealth interventions and those who did not. This comparative analysis offers valuable insights into the factors influencing the adoption or rejection of telehealth services among older populations. Reasons for not attending or continuing with face-to-face programs for the prevention of falls have been identified, such as younger–older people who consider that the intervention is targeted at more frail or sicker older people and/or prefer more active activities [41] and lack of motivation to perform the exercises on their own at home (due to laziness or distractions) or not feeling confident in their abilities to perform them, which affects their attendance or the implementation of recommendations [44]. This could partly explain and/or is in accordance with some reasons identified in our study, such as the lack of motivation to perform the intervention alone, and in contrast, the positive evaluation of the support provided by the professionals mentioned as a facilitator with the intervention of telehealth.

Considering that older people in the IG maintained biopsychosocial effects from the in-person modality and established a new routine with telehealth (turning it into a coping strategy during the pandemic) and their mood was not affected during the pandemic, several psychosocial factors can be highlighted as underlying the facilitators and barriers identified in this research. These factors may explain the commitment and motivation of older people to continue or not with the asynchronous telehealth intervention, such as health self-efficacy, health literacy, healthy identities, perceived health, control beliefs in activity, social support, and psychological well-being. These aspects could be further explored and enhanced in future research and telehealth interventions with older people to promote participation in such initiatives. It has been reported that health literacy and health self-efficacy are significantly and positively associated, and they mediate online communication between providers and older people in the use of digital technologies, electronic medical records, and telemedicine [45]. Similarly, these factors are associated with the quality of life of older people, influencing aspects such as sleep quality, fruit and vegetable consumption, exercise, sedentary behavior, and psychological health [45]. Additionally, it is suggested that the provider–older adult relationship plays an important role in their quality of life [45].

It has also been described that health efficacy, linked to a stronger health identity (valuing one's health highly), is a predictor of psychological well-being in middle and older age [46]. Similarly, self-efficacy and self-esteem have been shown to promote physical, psychological, and social well-being, making it essential to encourage older people to participate in physical, recreational, social, and cognitive activities, thereby enhancing their overall well-being [47].

Prior to the pandemic, a personalized approach to motivation and behavior change was proposed to achieve sustained behavioral changes in physical activity among adults and older people. This approach includes social support, goal setting, and positive effects, along with cognitive restructuring of negative and self-destructive attitudes and misconceptions [48]. These strategies can lead to increased self-efficacy and control beliefs regarding exercise, as well as self-management skills such as self-regulation and action planning. In turn, these improvements contribute to long-term increases in physical activity, physical well-being, psychological well-being, and overall quality of life [48].

During the pandemic, active and healthy older people who participated in community groups exhibited positive socioemotional indicators related to well-being, such as self-efficacy, social support, perceived health, and proactive behavior. These factors helped them face the pandemic and cope with daily stressors [49]. This highlights the importance of coping strategies and social participation in enhancing older people's ability to mitigate the psychological consequences of crises, further providing evidence for the benefits of aging in place [49].

The manual and the videos were greatly appreciated by older people from both groups. However, older people from the CG mentioned that they received it but did not use it since they did not have someone to help them. Therefore, it could be hypothesized that the manual as a single intervention tool is less effective, compared to videos or a combination of both. A deficit in our telehealth intervention was not conducting more frequent support strategies or telephone follow-ups. A strategy that could enhance the delivery of the manual and follow-ups/support is based on telehealth research conducted during the pandemic [16], which included a manual with an activity calendar with scheduled dates, for older people to mark them, so they self-reported the number of completed sessions during phone calls; motivational, psychological, and practical elements were also incorporated, together with the setting of goals and with a focus on independence, giving older people the option of choosing the physical activity of their preference, which favored adherence and empowerment. This might enable the establishment of positive user–professional relationships, the resolution of doubts, health and intervention-related monitoring, and the creation of self-efficacy in older people.

Consequently, when introducing new ICTs, the steep learning curve older people may experience must be recognized, and support should be offered to help them become familiar with technologies [15]. Additionally, they may feel uncomfortable, lack access to the necessary technology, and/or have sensory difficulties (impaired hearing or vision), hampering their use [17]. For this reason, it is recommended to include scales that assess the usability and/or proficiency in cell phone/tablet/Internet, such as the Mobile Device Proficiency Questionnaire [50], which may be useful to facilitate training of older people, applying the teaching–learning principles of adult training.

In addition, perceptions of older people toward ICTs should be identified to promote their use, such as intrinsic factors: attitudes around control, independence, and the need or requirements for safety, and extrinsic factors: usability, feedback, and costs. In addition, to prevent rejection or withdrawal, technologies should adapt to older people's lifestyles and expectations and offer tailored interventions that take into account their preferences and capabilities [15], and ICTs' positive benefits should be emphasized aimed at encouraging older people's independence [51]. To the extent that older people are more informed and empowered with their care, they will be able to carry out interventions in the telehealth modality.

It will be important to move toward telehealth interventions addressing the diversity and particular needs of older people [6], differentiating by sex, age, functionality, education level, digital literacy, and context. Regarding the design of regular multicomponent (physical activity + cognitive stimulation + education) telehealth interventions, exercises included in the physical activity component should be prescribed according to the FITT principles (frequency, intensity, time, and type of exercise), going beyond the recommendations of WHO [23]; concerning cognitive stimulation, the stimulation of several higher functions and awareness of holistic factors including older people's perceptions should be incorporated [52]; and in the education component, the promotion of habits regarding physical activity, nutrition, sleep, alcohol, mental health, and mental and social activities should be included as minimum features [53]; in accordance with the experience found in our study, follow-ups, the inclusion of family members, and adherence should also be promoted.

In a pandemic context, multicomponent telehealth interventions should incorporate physical activity, cognitive stimulation, and emerging measures of social prescription against mood affectation and loneliness [1]. The present study focused on the intervention in asynchronous telehealth (videos, infographics, and manual sent via WhatsApp); even previous findings have shown that WhatsApp usage reduced loneliness in older people [54]. In cases in which telehealth could not be definitely implemented, support strategies for older people should be developed, whether they are provided via telephone, social networks, or home visits, regularly carrying infographics, guidelines, and/or manuals for its performance and subsequent feedback [5].

In the future, research on multicomponent interventions in asynchronous telehealth modality targeted at older people should be further explored, whether they are studied as single interventions or compared to synchronous and/or face-to-face modalities. Additionally, it is important to compare the barriers and facilitators among groups of older people with different contexts (urban and rural), sociodemographic factors, health-related factors, levels of health literacy, and digital literacy. Similarly, motivation and knowledge of technologies (cell phone, computer/tablet, and Internet) should be assessed within the context of Comprehensive Gerontological Assessment [55]. In addition, ICTs' literacy should be incorporated as a prior intervention being conducted telehealth, to prevent rejection or withdrawal, considering also that specific educational strategies about telehealth for low-income older people have been shown to increase confidence in accessing and using telehealth in the future, while also enhancing the likelihood of health promotion among them [56], and reduces digital inequalities in access to health services.

Our results allowed us to identify, from the perspective of the users and based on the effective coverage model [20], facilitators and barriers in accessibility, acceptability, contact, and effectiveness coverage, obtaining information from those who continued with telehealth and those who did not, which enriches the information to date in this field of study.

Weaknesses of the present study include little monitoring and follow-up work, which finally resulted in a smaller group participating in telehealth intervention, compared to the group that did not take part. Considering the confinement of older people during the pandemic, the only way to conduct evaluations was through telephone calls. As a result, it is possible that some older people may not have provided in-depth reflections but rather socially acceptable responses. Therefore, we believe that in the future, it is important to include video calls and/or objective evaluations using assessment tools within the context of the Comprehensive Gerontological Assessment [57]. Strengths include cost-effective and replicable intervention protocol, professional training, duration of telehealth intervention, sample size, and in-depth interviews that enabled the identification of diverse facilitators and barriers.

## 5. Conclusions

This is the first qualitative study that identifies facilitators and barriers of a promotional–preventive multicomponent intervention in an asynchronous telehealth modality, as a continuation of the same face-to-face intervention, and provides valuable perspectives on the design and implementation and lessons learned for future interventions with the context of public policy. Asynchronous telehealth could be used regularly with older people in rural areas and in situations of disability and/or with care needs, pandemic scenarios, or natural disasters, managing these facilitators and barriers to enable access and equity in health. Future research may benefit from the evaluation of other types of telehealth.

## Figures and Tables

**Table 1:** Characteristics of the parameters of the multicomponent intervention (physical activity, cognitive stimulation, and education) in the asynchronous telehealth modality carried out by the elderly in the intervention group.

**Intervention group (28)**					
	***n***	**Mean**	**SD**	**Min**	**Max**

*Physical activity*					
Frequency (days per week)		2.68	1.63	1	7
Duration (activity time in minutes)		32.32	14.43	10	60
Type of exercise (yes)					
Cardiovascular aerobic (%)	22	78.57			
Upper limb strength (%)	15	53.57			
Lower limb strength (%)	24	85.71			
Upper limb flexibility (%)	25	89.29			
Lower limb flexibility (%)	27	96.43			
Balance (%)	26	92.86			
Exercise intensity					
Light (%)	7	25.00			
Moderate (%)	19	67.86			
Intense (%)	2	7.14			

*Cognitive stimulation*					
Frequency (days per week)		2.04	1.20	1	5
Duration (activity time in minutes)		26.30	14.35	5	60

*Education*					
Frequency (days per week)		2.18	1.94	1	7
Duration (activity time in minutes)		27.73	14.87	10	60

**Table 2:** Description of older people from the intervention group who participated in the multicomponent intervention in the asynchronous telehealth modality and of older people from the control group who did not participate.

Interview number	Sex	Age	Level of education	Years participating in the program
*Intervention group*				
1	Female	73	Complete secondary education	4
2	Female	66	Incomplete secondary education	4
3	Female	75	Incomplete primary education	2
4	Female	80	Complete primary education	3
5	Female	69	Complete secondary education	2
6	Female	80	Incomplete secondary education	6
7	Female	60	Incomplete higher education	2
8	Female	68	Complete higher education	3
9	Male	72	Incomplete higher education	2
10	Male	67	Incomplete higher education	3
11	Male	75	Complete higher education	1
12	Female	69	Incomplete secondary education	4
13	Female	65	Complete higher education	4
14	Female	60	Incomplete higher education	2

*Control group*				
1	Female	73	Incomplete primary education	2
2	Male	79	Incomplete secondary education	4
3	Male	88	Complete primary education	3
4	Female	62	Complete secondary education	1
5	Female	75	Incomplete secondary education	4
6	Female	73	Complete secondary education	1
7	Male	79	Complete secondary education	5
8	Female	72	Complete primary education	5
9	Female	71	Complete higher education	4
10	Female	69	Incomplete secondary education	3
11	Female	68	Complete secondary education	2
12	Male	73	Complete higher education	2

## Data Availability

Data are available upon request from the corresponding author (rafael.pizarro@uss.cl).

## Supporting Information

Consult as Supporting 1, the consolidated criteria for reporting qualitative research (COREQ) 32-item checklist this a study.
