# Supplementary material for: Perceived Facilitators and Barriers, From the Perspective of Users, of a Multicomponent Intervention in Older People Using an Asynchronous Telehealth Modality During the COVID-19 Pandemic: A Qualitative Research
Source: J Aging Res. 2025 Mar 31;2025:6839569. doi: 10.1155/jare/6839569 (PMC11976052; doi:10.1155/jare/6839569)
Supplement: Supporting Information — Additional supporting information can be found online in the Supporting Information section. [file 6839569.f1.docx]

**Supplementary 1. Consolidated criteria, COREQ 32-item checklist.**

| **No. Item** | **Guide questions/description** | **Reported on Page #** |
| --- | --- | --- |
| **Domain 1: Research team and reﬂexivity** |  |  |
| *Personal Characteristics* |  |  |
| 1. Inter viewer/facilitator | Which author/s conducted the interview or focus group? | **Principal Investigator** |
| 2. Credentials | What were the researcher’s credentials? E.g. PhD, MD | **Physical Therapist. PhD in Gerontology Research, Master’s degree in Social Gerontology and Master’s degree in Health Management and Direction** |
| 3. Occupation | What was their occupation at the time of the study? | **Lectureship at the University** |
| 4. Gender | Was the researcher male or female? | **Man** |
| 5. Experience and training | What experience or training did the researcher have? | **More than 20 years of working and teaching undergraduate and graduate courses in Gerontology** |
| *Relationship with participants* |  |  |
| 6. Relationship established | Was a relationship established prior to study commencement? | **Yes. Only When the Research was explained. In the signature of the informed consent, 10 months before the interviews. The researcher had no interference in the Telehealth intervention itself** |
| 7. Participant knowledge of the interviewer | What did the participants know about the researcher? e.g. personal goals, reasons for doing the research | **Participants were briefed on the purpose of the study and understood it. Participants reviewed the participant information documentation prior to giving their written informed consent to be involved, which was approved for our ethical committee** |
| 8. Interviewer characteristics | What characteristics were reported about the inter viewer/facilitator? e.g. Bias, assumptions, reasons and interests in the research topic | **His training (PhD in Gerontological Research) and experience (More than 20 years of working and teaching undergraduate and graduate courses in Gerontology)** |

| **Domain 2: study design** |  |  |
| --- | --- | --- |
| *Theoretical framework* |  |  |
| 9. Methodological orientation and Theory | What methodological orientation was stated to underpin the study? e.g. grounded theory, discourse analysis, ethnography, phenomenology, content analysis | **The technique of in-depth semi-structured interviews was used. Thematic analysis was used to report patterns** |
| *Participant selection* |  |  |
| 10. Sampling | How were participants selected? e.g. purposive, convenience, consecutive, snowball | **Intentional sample** |
| 11. Method of approach | How were participants approached? e.g. face-to-face, telephone, mail, email | **Telephone interview** |
| 12. Sample size | How many participants were in the study? | **79 older people started the intervention and 73 were eligible.**  **Finally, 26 older people answered the interviews** |
| 13. Non-participation | How many people refused to participate or dropped out? Reasons? | **6 older people dropped out. None refused to participate** |
| *Setting* |  |  |
| 14. Setting of data collection | Where was the data collected? e.g. home, clinic, workplace | **The older people were at their home at the time of the interviews** |
| 15. Presence of non-participants | Was anyone else present besides the participants and researchers? | **No. Only the researcher and the interviewee were present.** |
| 16. Description of sample | What are the important characteristics of the sample? e.g. demographic data, date | **They are described in the table 1** **(sex, age, schooling, seniority in the program)** |
| *Data collection* |  |  |
| 17. Interview guide | Were questions, prompts, guides provided by the authors? Was it pilot tested? | **A semi-structured in-depth interview guide was developed. The guidelines were based on existing literature and related to the effects, barriers, and facilitators of Telehealth and multicomponent interventions in older people** |
| 18. Repeat interviews | Were repeat inter views carried out? If yes, how many? | **They were unique interviews** |
| 19. Audio/visual recording | Did the research use audio or visual recording to collect the data? | **The interviews were audio recorded** |
| 20. Field notes | Were ﬁeld notes made during and/or after the inter view or focus group? | **The main researcher took field notes after the interviews** |
| 21. Duration | What was the duration of the inter views or focus group? | **The interviews lasted between 30-60 minutes** |
| 22. Data saturation | Was data saturation discussed? | **Theoretical saturation was performed** |
| 23. Transcripts returned | Were transcripts returned to participants for comment and/or correction? | **Yes. The transcripts were returned to participants for comment and/or correction** |
| **Domain 3: analysis and ﬁndings** |  |  |
| *Data analysis* |  |  |
| 24. Number of data coders | How many data coders coded the data? | **A sociologist researcher, together with 3 researchers** |
| 25. Description of the coding tree | Did authors provide a description of the coding tree? | **A coding tree was developed** |
| 26. Derivation of themes | Were themes identiﬁed in advance or derived from the data? | **An inductive approach was used in analysis with data-driven codes and themes. The meanings resulted in themes and sub-themes based on the statements of the older people** |
| 27. Software | What software, if applicable, was used to manage the data? | **Atlas.Ti** |
| 28. Participant checking | Did participants provide feedback on the ﬁndings? | **Y**es. **They were socialized** |
| *Reporting* |  |  |
| 29. Quotations presented | Were participant quotations presented to illustrate the themes/ﬁndings? Was each quotation identiﬁed? e.g. participant number | **Introductory quotes were identified that reflect the vast majority of participants. The group, interview number, sex, age and seniority in the program were identified** |
| 30. Data and ﬁndings consistent | Was there consistency between the data presented and the ﬁndings? | **Yes. Their consistency was between the data presented and the ﬁndings** |
| 31. Clarity of major themes | Were major themes clearly presented in the ﬁndings? | **Yes. Major themes clearly were presented in the ﬁndings** |
| 32. Clarity of minor themes | Is there a description of diverse cases or discussion of minor themes? | **Yes. Diverse cases were described, and minor themes were discussed.** |
